# Correction: Audrito et al. NAMPT Over-Expression Recapitulates the BRAF Inhibitor Resistant Phenotype Plasticity in Melanoma. *Cancers* 2020, *12*, 3855

**DOI:** 10.3390/cancers18152426

**Published:** 2026-07-28

**Authors:** Valentina Audrito, Vincenzo Gianluca Messana, Enrico Moiso, Nicoletta Vitale, Francesca Arruga, Lorenzo Brandimarte, Federica Gaudino, Elisa Pellegrino, Tiziana Vaisitti, Chiara Riganti, Roberto Piva, Silvia Deaglio

**Affiliations:** 1 Cancer Immunogenetics Lab, Department of Medical Sciences, University of Turin, 10126 Turin, Italy; vincenzo.messana@unito.it (V.G.M.); nicoletta.vitale@unito.it (N.V.); francesca.arruga@unito.it (F.A.); lorenzo.brandimar@edu.unito.it (L.B.); federica.gaudino@unito.it (F.G.); tiziana.vaisitti@unito.it (T.V.); 2 Koch Institute for Integrative Cancer Research, Massachusetts Institute of Technology, Cambridge, MA 02139, USA; emoiso@mit.edu; 3 Broad Institute of Harvard and MIT, Cambridge, MA 02142, USA; 4 Department of Molecular Biotechnologies and Health Sciences, University of Turin, 10126 Turin, Italy; elisa.pellegrino@unito.it (E.P.); roberto.piva@unito.it (R.P.); 5 Department of Oncology, University of Turin, 10126 Turin, Italy; chiara.riganti@unito.it

## Error in Figure

In the original publication [1], there was a mistake in Figure 6A as published. The discrepancy identified in Figure 6A derives from an inadvertent figure assembly error during preparation of the cropped Actin panel for the A375/BiR TTA cells. Specifically, an incorrect crop was mistakenly inserted in the final assembled figure, whereas the original full-length unedited blot already provided in the Supplementary Information corresponds to the correct experiment and underlying raw data.

The corrected Figure 6A (original Actin for A375/BiR TTA) appears below. The authors state that the scientific conclusions are unaffected. This correction was approved by the Academic Editor. The original publication has also been updated.


