# Supplementary material for: NAMPT Over-Expression Recapitulates the BRAF Inhibitor Resistant Phenotype Plasticity in Melanoma
Source: Cancers (Basel). 2020 Dec 20;12(12):3855. doi: 10.3390/cancers12123855 (PMC7766175; doi:10.3390/cancers12123855)
Supplement: Supplementary file 1 [file cancers-12-03855-s001.pdf]

cancers

Supplementary Materials: NAMPT Over-Expression Recapitulates the BRAF Inhibitor Resistant Phenotype Plasticity in Melanoma

Valentina Audrito, Vincenzo Gianluca Messana, Enrico Moiso, Nicoletta Vitale, Francesca Arruga, Lorenzo Brandimarte, Federica Gaudino, Elisa Pellegrino, Tiziana Vaisitti, Chiara Riganti, Roberto Piva and Silvia Deaglio

Text S1: Supplementary Methods

Reagents

Nicotinamide mononucleotide (NMN), Doxycycline (DOX) and Crystal-violet were from Sigma (Milan, IT). Vemurafenib was from Selleckchem (Munich, Germany).

Antibodies Used for Western Blot

The following antibodies were used for western blot: anti-NAMPT (A300-779A, Bethyl Laboratories, Montgomery, TX, USA), anti-phospho-ERK1/2 (pT202/pY204, #612359), anti-pan-ERK1/2 (#610124) both from BD Biosciences (San Jose, CA, USA). anti-Twist (Twist2C1a, sc-81417), anti-ZEB1 (H-3, sc-515797), anti-Vimentin (sc-6260), anti-β-Actin (sc-47778) and anti-Vinculin (H-10, sc-25336) all from Santa Cruz Biotechnology (Dallas, TX, USA). Secondary reagents were: goat anti-mouse IgG-HRP-conjugated (Perkin Elmer, Monza, IT) and goat anti-rabbit HRP-conjugated (Santa Cruz Biotechnology).

Antibodies Used for FACS Analysis

The following antibodies were used for FACS: anti-CD56/NCAM Brilliant Violet 750™ (#362555, BioLegend, San Diego, CA, USA), CD133-APC (#17-1339-42), CD49c/Integrinα3-APC (#17-0494-42) all from eBioscience (Milan, IT).

Antibodies Used for Confocal Microscopy

Antibodies used for confocal microscopy were: anti-GLUT1 (ab652, 1:500, Abcam), and anti-ZEB1 (H-3, 1:50, sc-515797), anti-Vimentin (1:50, sc-6260), anti-MRP1 (QCRL-1, 1:50, sc-18835) all from Santa Cruz Biotechnology. Secondary reagents were: goat anti-mouse IgG AlexaFluor647-conjugated (1:100) and goat anti-rabbit IgG AlexaFluor647-conjugated (1:100), both from Thermo Fisher (Monza, IT).

Cell Culture

Cell lines were cultured in RPMI-1640 (Sigma, Milan, IT) or Dulbecco's Modified Eagle Medium (DMEM, from Thermo Fischer Scientific, Monza, IT) supplemented with 10% of fetal calf serum (FCS), and 10 IU/mL of penicillin / streptomycin (all from Sigma). BRAF inhibitor (BRAFi)-resistant cells were generated as specified in [1] and indicated as /BiR cells. /BiR cells were maintained in culture in complete medium, adding BRAFi at the concentration of 1.6 μM (dabrafenib, GlaxoSmithKline, Brentford, UK). In the experiment with NMN, cells were treated with NMN 1 mM for 24 hours, added at the beginning of treatment.

Inducible NAMPT Over-Expression

To obtain doxycycline-dependent inducible NAMPT over-expression pCW57.1-NAMPT-GFPtag vector was constructed by replacing the Cas9 of the pCW57.1-Cas9 vector (Addgene, Watertown, MA, USA plasmid # 50661) with the NAMPT-GFPtag cassette from

pRRL.sin.PPT.hCMV.NAMPT-GFPtag.WPRE vector, using the NheI-BamHI sites. Vector stocks were produced by transient transfection of the transfer plasmid, the packaging plasmids pMDLg/pRRE and pRSV.REV, and the vesicular stomatitis virus (VSV) envelope plasmid pMD2.VSV-G (2.5, 1.0, 0.5, and 0.6 μg, respectively, for 10 cm dishes) in 293T cells in the presence of Effectene (Qiagen, Milan, IT), following the manufacturer's instructions. Stable cell lines expressing the construct were selected by treatment with 2 μg/mL puromycin (Sigma-Aldrich, Milan, IT) for 24 hours. NAMPT-GFPtag expression was induced by doxycycline treatment (1 μg/mL) for 24 hours and then GFP + cells were flow sorted (FACSAriaIII, BD Biosciences, San Jose, CA, USA) and expanded.

Inducible NAMPT Silencing (shRNA NAMPT)

pLVTHM-GFP-shRNA vectors were constructed by subcloning the U6 promoter–shRNA-NAMPT and shRNA-CTRL cassette into the EcoRI-ClaI sites of the pLVTHM vector, kindly provided by D. Trono (University of Geneva, Geneva, Switzerland) [2]. For conditional RNAi, A375/BiR and M14/BiR MM cell lines were transduced at high efficiency with pLV-DsRed-tTRKRAB plasmid, expanded, and used for transduction with pLVTHM-GFP-shRNA-NAMPT or pLVTHM-GFP-shRNA-CTRL lentiviral particles. Next, cells were treated with doxycycline (1 μg/mL) for 24 hours to induce shNAMPT expression and double GFP+/DsRed+ cells were flow sorted (FACSAriaIII, BD Biosciences) and expanded.

CRISPR-CAS9 NAMPT Knock-Out (KO) Cells

CRISPR/Cas9 NAMPT/KO was prepared as previously published [3]. Briefly, the pX458 vector expressing Cas9 and a GFP tag [pSpCas9(BB)-2A-GFP] was used (Addgene). RNA guides were designed to target NAMPT at exon 1 to generate a KO model and selected with the online CRISPR Design Tool (https://chopchop.cbu.uib.no). Sequences of the selected sgRNA: #2 TTGAACTCGGCTTCTGCCGC (exon1) and #7 AGCCGAGTTCAACATCCTCC (exon1). To clone the sgRNAs into the pX458 vector, two complementary oligos for each sgRNA were designed. After a denaturation/reannealing step, the two oligos were ligated with the linearized pX458, digested with BpiI (New England Biolabs, USA). The ligated DNA reaction underwent a plasmid SAFE digestion, to eliminate linearized DNA. The 7 uL of SAFE DNA reaction was used for bacterial transformation (25 uL of One Shot Stbl3 Chemically Competent E. Coli by Thermo Fisher). DNA insertion was done by thermal shock 30 sec at 42 °C and 2 min at 4 °C. After colonies formation, the correct insertion of the sgRNA sequences was confirmed by Sanger sequencing (Eurofins Genomics, Ebersberg, DE).

1μg of Cas9-GFP vectors containing the desired sgRNA (#2 and #7) was transfected into 5 × 105 A375/BiR cells using the Effectene® Transfection Reagent (Qiagen), following manufacturer's instruction. The Cas9-GFP vector without sgRNA was used as control (empty-vector). 24–30 hours after transfection, GFP + cells were sorted (FACSAriaIII, BD Biosciences) and cultured for functional experiments. In parallel, genomic DNA was extracted and used to check Cas9 cut efficiency with the Surveyor assay [3]. Validation of NAMPT silencing was obtained by Western blotting.

Quantitative Real-Time PCR (qRT-PCR) Primers

TaqMan Gene Expression Assays (Thermo Fisher Scientific) used are: Hs00237184_m1 (*NAMPT*), Hs00944470_m1 (*NMRK1*), Hs00376971_g1 (*NAPRT*), Hs00204757_m1 (*QPRT*), Hs01566408_m1 (*ZEB1*), Hs00361186_m1 (*TWIST*), Hs00185584_m1 (*VIMENTIN*), Hs00941830_m1 (*NCAM*), Hs00219905_m1 (*ABCC1*), Hs00892681_m1 (*SLC2A1*). Actin was used as housekeeping gene: Hs99999903_m1 (*ACTB*).

Western Blot Analysis

Western blot chemioluminescence reactions were visualized with ECL (Bio-Rad, Segrate, IT) using ImageQuant LAS4000 (GE Healthcare, Milan, IT) or ChemiDoc™ Touch Imaging System

(Bio-Rad). Densitometric analyses performed using ImageQuantTL 7.0 software (GE Healthcare) Image Lab 6.0.1 Software (Bio-Rad) or ImageJ/Fiji (https://imagej.net/Fiji). Total proteins were normalized over actin levels while phosphorylated protein over the corresponding non-phosphorylated protein levels.

Seahorse Metabolic Experiments

Oxygen consumption rate (OCR) and extracellular acidification rate (ECAR) were measured on 35,000 cells using Mito Stress Test Kit following the manufacturer's protocol and standard concentrations of oligomycin (1 μM), carbonylcyanide-4-(trifluoromethoxy)-phenylhydrazone (FCCP, 1 μM) and Rotenone/Antimycin A (0.5 μM). ECAR was measured using Glycolysis Stress Test kit, in basal conditions and in response to glucose (10 mM), oligomycin (1 μM) and 2-Deoxy-D-glucose (2-DG, 50 mM). All reagents were from Agilent. Data are the mean ± SEM from 3 separate experiments, with at least 4 replicates/sample. Results were analyzed using WAVE software (version 2.6.1, Agilent), normalized on cellular protein concentration (OD), and processed through the Mito Stress Test Report and Glycolysis Stress Test Report Generator (Agilent).

Confocal Microscopy

Before staining, coverslips were rinsed, fixed with 4% paraformaldehyde (PFA, 10 minutes, room temperature RT), permeabilized with 0.1% saponin in PBS (20 minutes, RT) and saturated with pre-immune goat serum (1 hour, 4 °C). Cells were then stained with the indicated primary and secondary antibodies and counter-stained with AlexaFluor 568-conjugated phalloidin (1:100) and 4',6-Diamidino-2-phenylindole (DAPI, 1:30,000, both from Thermo Fisher Scientific). Fluorescence was acquired by confocal microscopy, using an oil immersion 63× objective. Slides were analyzed using a TCS SP5 laser scanning confocal microscope; images were acquired with LAS AF software (both from Leica Microsystems, Milan, IT). Files were processed with Photoshop (Adobe Systems, San Jose, CA). Pixel intensity was calculated using the ImageJ software (http://rsbweb.nih.gov/ij/).

Statistics

Statistical TCGA analyses were performed using the R language (http://www.r-project.org), Rstudio (http://www.rstudio.com/). Correlations between NAMPT expression levels and other genes have been calculated by means of Pearson correlation coefficient.

Gene Set Enrichment Analysis (GSEA) analysis

TCGA SKCM cohort has been divided in high and low expressing samples based on median NAMPT expression levels. Analysis of differentially expressed genes (DEGs) between two clusters has been performed by means of limma [4] Bioconductor (http://www.bioconductor.org/) package, with standard settings. GSEA [5,6] was performed by running the GSEAPreranked tool from command line (gsea2-2.2.0.jar, with the following parameters: -mode Max_probe,-norm meandiv,-nperm 1000, -rnd_seed timestamp,-set_max 500 and-set_min 15). The Log2(FC) between NAMPT high and low samples has been used as ranking metric. The gene sets and the gsea jar file used in the analysis were downloaded from the Broad Institute GSEA website (http://software.broadinstitute.org/gsea/index.jsp), MSigDB database v6.0.

References

1. Audrito, V.; Managò, A.; La Vecchia, S.; Zamporlini, F.; Vitale, N.; Baroni, G.; Cignetto, S.; Serra, S.; Bologna, C.; Stingi, A.; et al. Nicotinamide Phosphoribosyltransferase (NAMPT) as a Therapeutic Target in BRAF-Mutated Metastatic Melanoma. *J. Natl. Cancer Inst.* **2018**, *110*, 290–303, doi:10.1093/jnci/djx198.
2. Wiznerowicz, M.; Trono, D. Conditional Suppression of Cellular Genes: Lentivirus Vector-Mediated Drug-Inducible RNA Interference. *J. Virol.* **2003**, *77*, 8957–8951, doi:10.1128/jvi.77.16.8957-8951.2003.
3. Arruga, F.; Gizdic, B.; Bologna, C.; Cignetto, S.; Buonincontri, R.; Serra, S.; Vaisitti, T.; Gizzi, K.; Vitale, N.; Garaffo, G.; et al. Mutations in NOTCH1 PEST domain orchestrate CCL19-driven homing of chronic lymphocytic leukemia cells by modulating the tumor suppressor gene DUSP22. *Leuk.* **2016**, *31*, 1882–1893, doi:10.1038/leu.2016.383.

4. Ritchie, M.E.; Phipson, B.; Wu, D.; Hu, Y.; Law, C.W.; Shi, W.; Smyth, G.K. limma powers differential expression analyses for RNA-sequencing and microarray studies. *Nucleic Acids Res.* **2015**, *43*, e47, doi:10.1093/nar/gkv007.
5. Subramanian, A.; Tamayo, P.; Mootha, V.K.; Mukherjee, S.; Ebert, B.L.; Gillette, M.A.; Paulovich, A.; Pomeroy, S.L.; Golub, T.R.; Lander, E.S.; et al. Gene set enrichment analysis: a knowledge-based approach for interpreting genome-wide expression profiles. *Proc. Natl. Acad. Sci. USA* **2005**, *102*, 15545–15550, doi:10.1073/pnas.0506580102.
6. Mootha, V.K.; Lindgren, C.M.; Eriksson, K.F.; Subramanian, A.; Sihag, S.; Lehar, J.; Puigserver, P.; Carlsson, E.; Ridderstrale, M.; Laurila, E., et al. PGC-1alpha-Responsive Genes Involved in Oxidative Phosphorylation Are Coordinately Downregulated in Human Diabetes. *Nat. Genet.* **2003**, *34*, 267–273, doi:10.1038/ng1180.

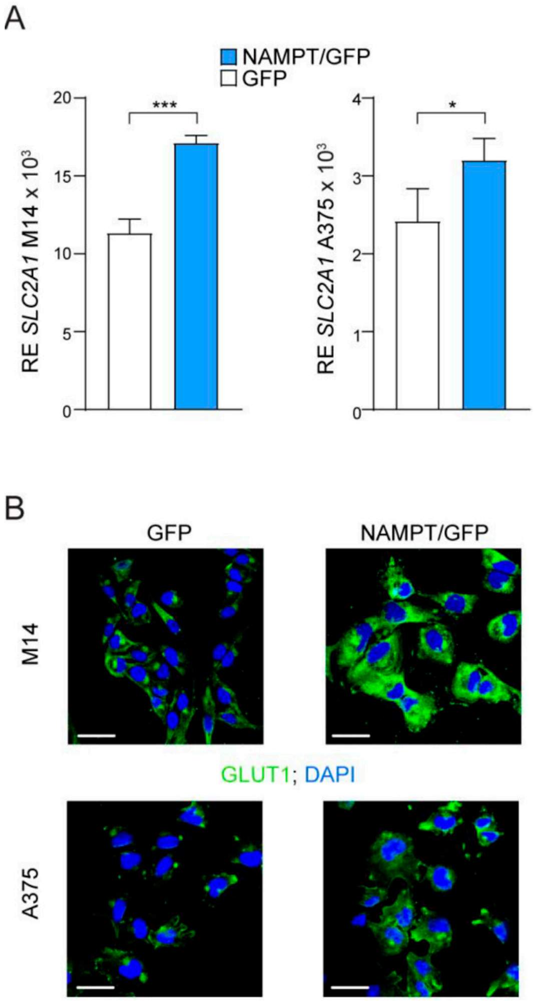

Figure S1. NAMPT over-expression increases glucose transporter expression. (**A**) Histograms reporting mRNA expression levels of the glucose transporter GLUT1/SLC2A1 in the indicated cell line variants. Expression levels of the analyzed gene were normalized over actin. Results were obtained from at least 5 independent experiments. Unpaired *t* test. (**B**) Confocal staining for GLUT1 (green fluorescence) in M14 and A375 cell lines comparing NAMPT/GFP and GFP variants (original magnification 63×, scale bar: 50 μm). Data in the Figure are presented as the mean ± SEM. Significance was represented as: * $p \leq 0.05$ and *** $p \leq 0.001$.

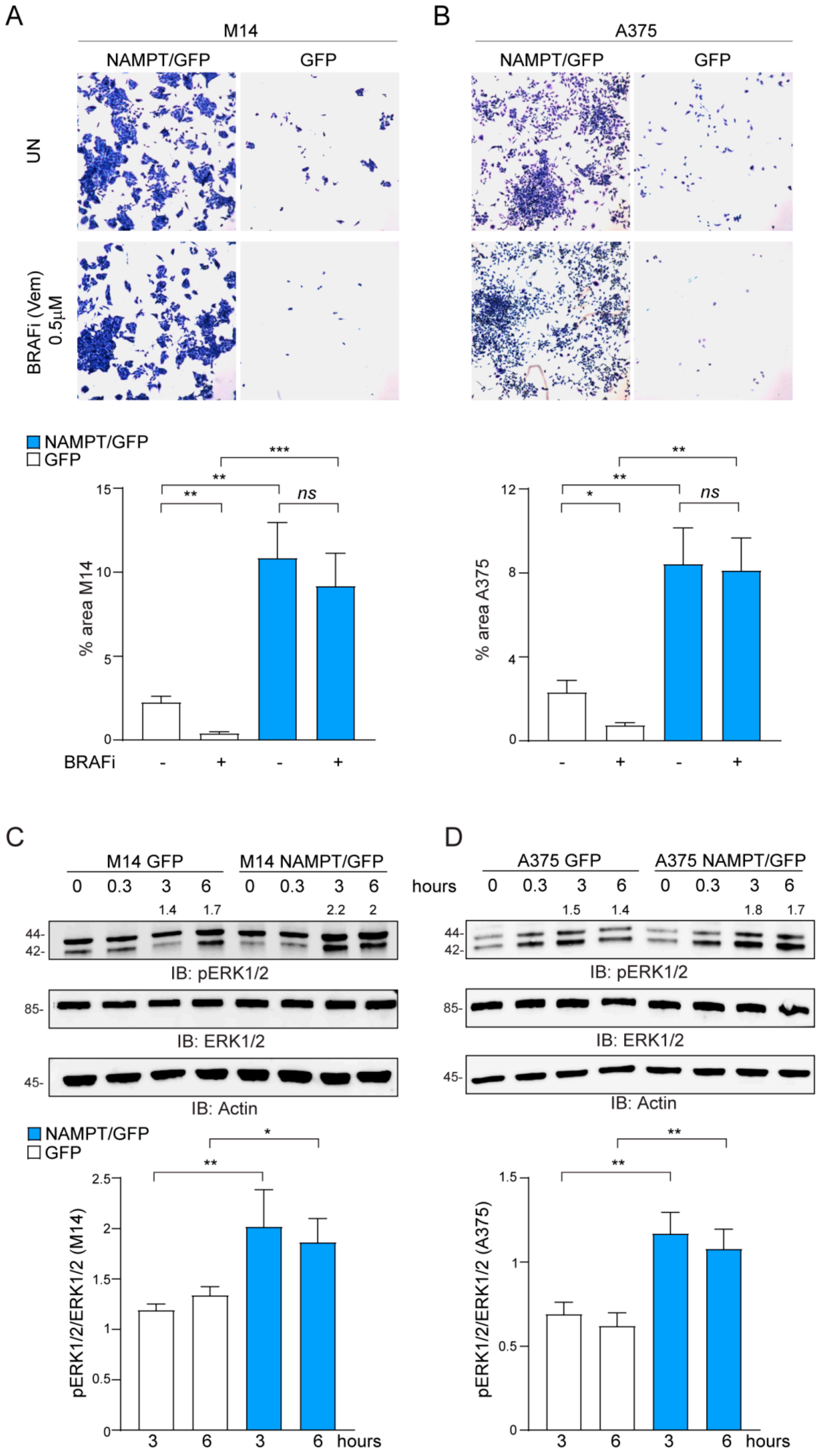

Figure S2. NAMPT over-expression supports growth and ERK1/2 phosphorylation. (A–B) Colony-forming ability of M14 (**A**) and A375 (**B**) NAMPT/GFP in comparison with GFP control cells in untreated (UN) or treated with the indicated dose of vemurafenib (BRAFi, 0.5 μM) every 48 h for 7

days. Cells were stained with crystal violet, and representative images (magnification 2.5×), acquired using Olympus microscope, are shown. Below the images, histograms show the cumulative quantification of the percentage (%) area with colonies at the end of the period (3 independent experiments performed in triplicates, Mann-Whitney and paired *t* test) (C–D) Cells were starved (24 hours) and then activated with serum for the indicated time points. Representative western blots for pERK1/2 and total ERK1/2 using whole cell lysates of M14 GFP and NAMPT/GFP (**A**) or A375 GFP and NAMPT/GFP (**B**). Actin was added as loading control. Densitometry intensity ratios of pERK1/2 bands over ERK1/2 were reported. Below the images, histograms show the cumulative quantification of pERK1/2 over ERK1/2 at 3 and 6 hours of serum activation in both cell lines (at least 5 independent experiments, Mann-Whitney test). Data in the Figure are presented as the mean ± SEM. Significance was represented as: * $p \leq 0.05$, ** $p \leq 0.01$, *** $p \leq 0.001$ and **** $p \leq 0.0001$. Uncropped Western Blot Images in Figure S12.

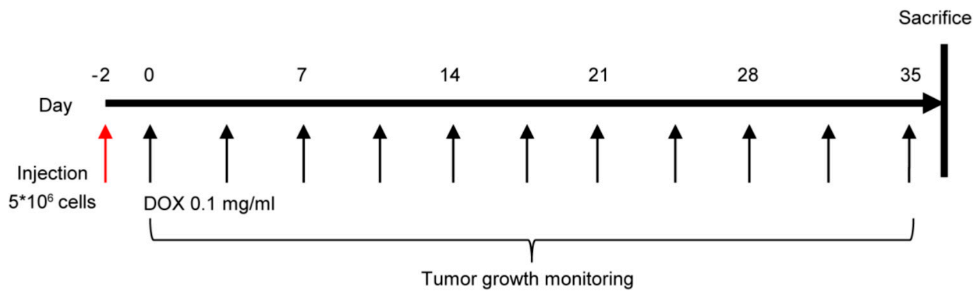

Figure S3. Mice treatment scheme. NSG mice were injected subcutaneously with NAMPT inducible A375 or M14 cells. After ~48 hours post-injection Doxycyclin (DOX 0.1 mg/mL) were administrated orally twice a week. After ~1 month mice were sacrificed and tumors analyzed. A group of mice without DOX treatment was used as control.

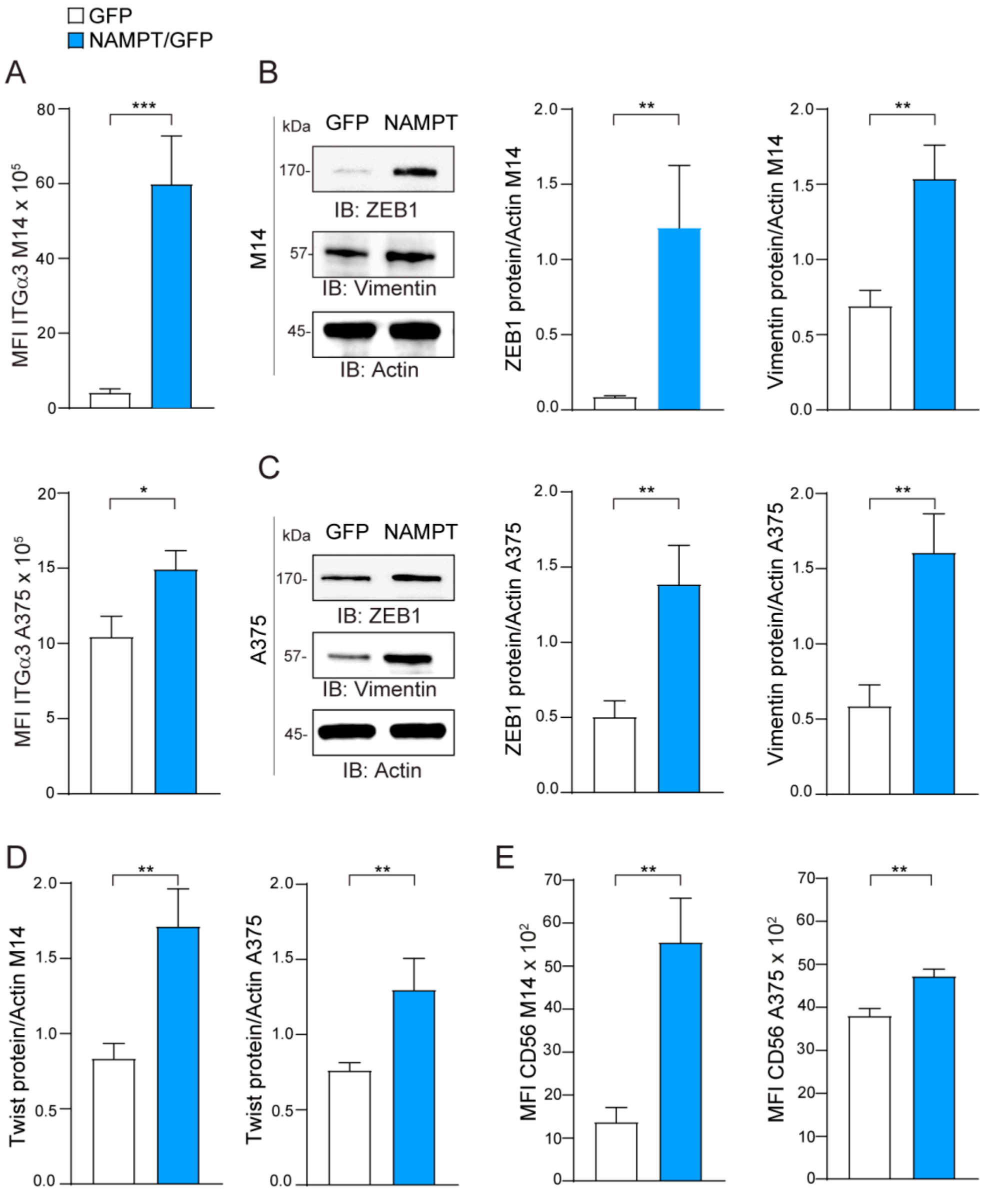

Figure S4. NAMPT over-expression forces EMT molecules expression. (**A**) Differential expression of integrin-α3 (ITGα3) in M14 and A375 NAMPT/GFP and GFP variants detected by FACS analysis. Cumulative data shows mean fluorescence intensity (MFI) of 6 independent experiments. Mann-Whitney test. (B–C) Representative western blot showing expression of mesenchymal molecules including ZEB1 and VIM. Histograms show cumulative data of band quantification, derived from at least 5 independent western blots, represented as ratio of the indicated protein / actin levels, in M14 (**B**) and A375 (**C**) variants. Mann-Whitney test. (**D**) Histograms show cumulative data of Twist protein band quantification, derived from at least 5 independent western blots, represented as ratio of the indicated protein/actin levels, in M14 and A375 variants. (E) Cumulative date show mean fluorescence intensity (MFI) of the differential expression of CD56 in M14 and A375 NAMPT/GFP and GFP variants detected by FACS analysis (6 independent experiments, Mann-Whitney test). Data in the Figure are presented as the mean ± SEM. Significance was represented as: * $p \leq 0.05$, ** $p \leq 0.01$, *** $p \leq 0.001$ and **** $p \leq 0.0001$.Uncropped Western Blot Images in Figure S13.

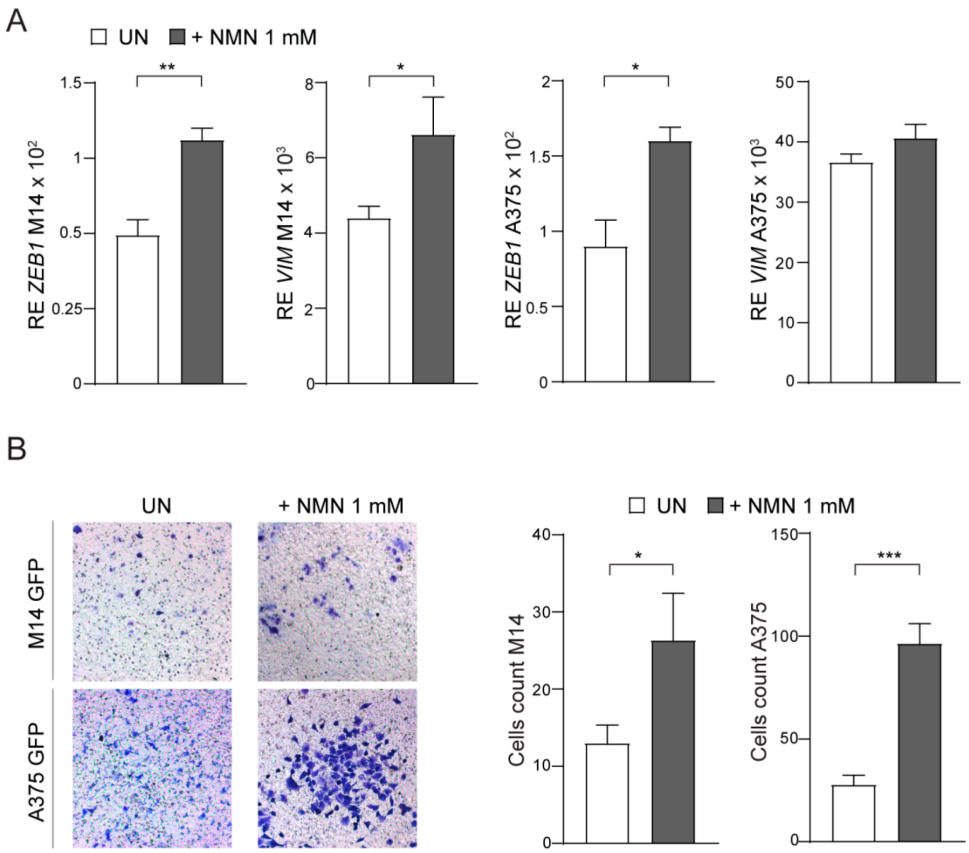

Figure S5. Effects of the addition of the NAMPT product NMN to GFP cells. (**A**) GFP cells were treated with NMN 1 mM for 24 hours and then RT-PCR was performed to check expression of ZEB1 and its target VIM. Results were obtained from 3 independent experiments. Unpaired *t* test. (**B**) Representative images (×10 magnification) of invasion assay in matrigel for 24 hours of M14 and A375 cell lines GFP variants in the presence or not of NMN 1 mM. Histograms on the right represent cumulative data of invasion assays (3 independent experiments). Unpaired *t* test. Data in the Figure are presented as the mean ± SEM. Significance was represented as: * $p \leq 0.05$, ** $p \leq 0.01$, *** $p \leq 0.001$ and **** $p \leq 0.0001$.

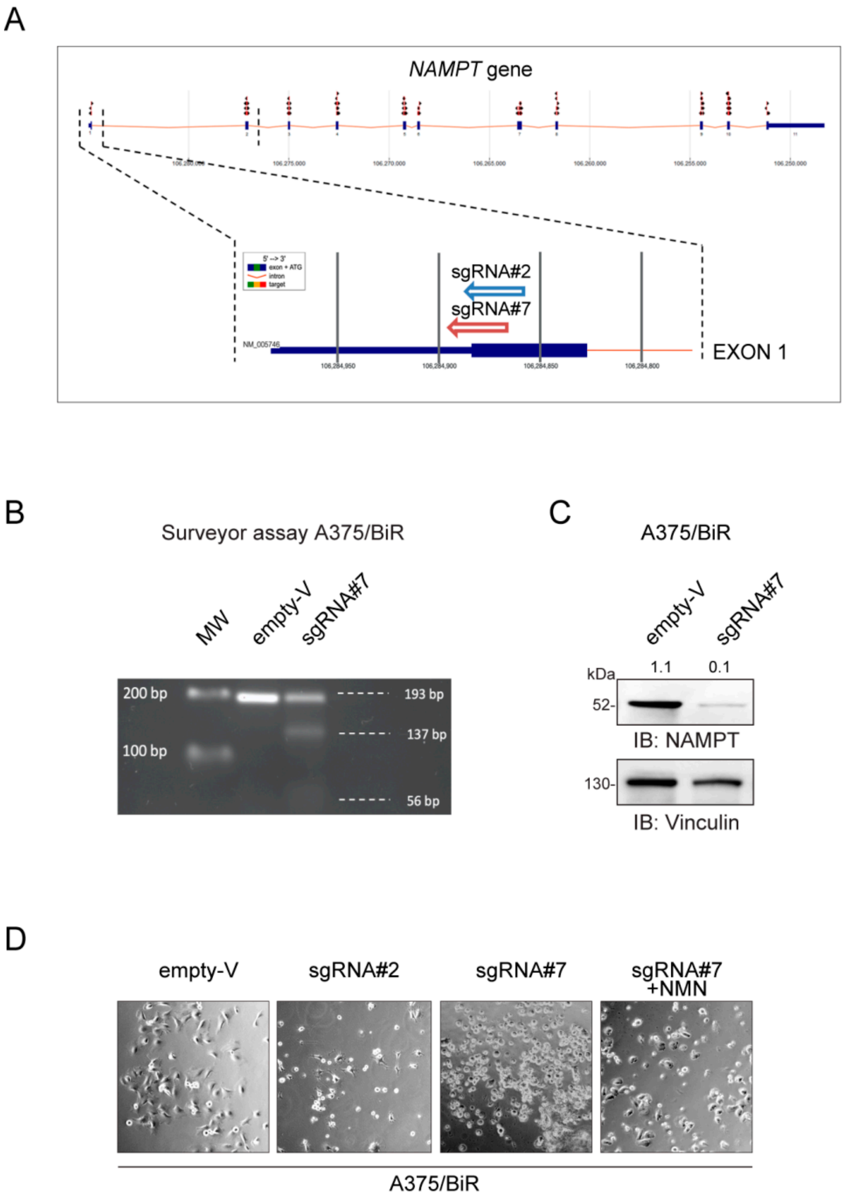

Figure S6. Full *NAMPT* knock-out (KO) using CRISPR/CAS9 technology. (**A**) Schematic representation of the selected two guides (sgRNAs) based on their specificity in targeting the first (#2, #7) exon of *NAMPT* gene. (**B**) The picture represents the surveyor assay analysis of DNA fragments of A375/BiR Cas9/Empty-V and A375/BiR Cas9/sgRNA#7. The designed region of interest was amplified obtaining a 193 base pair (bp) product in which the Cas9 cutting site falls (as visible in empty-V lane). Theoretical Double-Strand-Break (DBS) cut divides into two fragments of 137 bp and 56 bp respectively (as visible in sgRNA#7 lane). MW: molecular weight marker. (**C**) Images show the western blot for NAMPT of A375/BiR cells transfected with Cas9/sgRNA#7 and empty-V. Vinculin was used as loading control. (**D**) Images, acquired using Axio Observer Z1 (×20 magnification), showing A375/BiR cells sorted after 24 h from transfection with empty-V, Cas9/sgRNA#2 and Cas9/sgRNA#7 and left in culture for other 48–72 h. After 48 h *NAMPT*-KO cells start to die until 72 h in which they were completely detached from the wells. Control cells (empty-V) were not affected by transfection, as they show regular morphology in the picture. For Cas9/sgRNA#7nicotinamide mononucleotide (NMN, 0.5 mM) was added to rescue NAMPT depletion. Uncropped Western Blot Images in Figure S14.

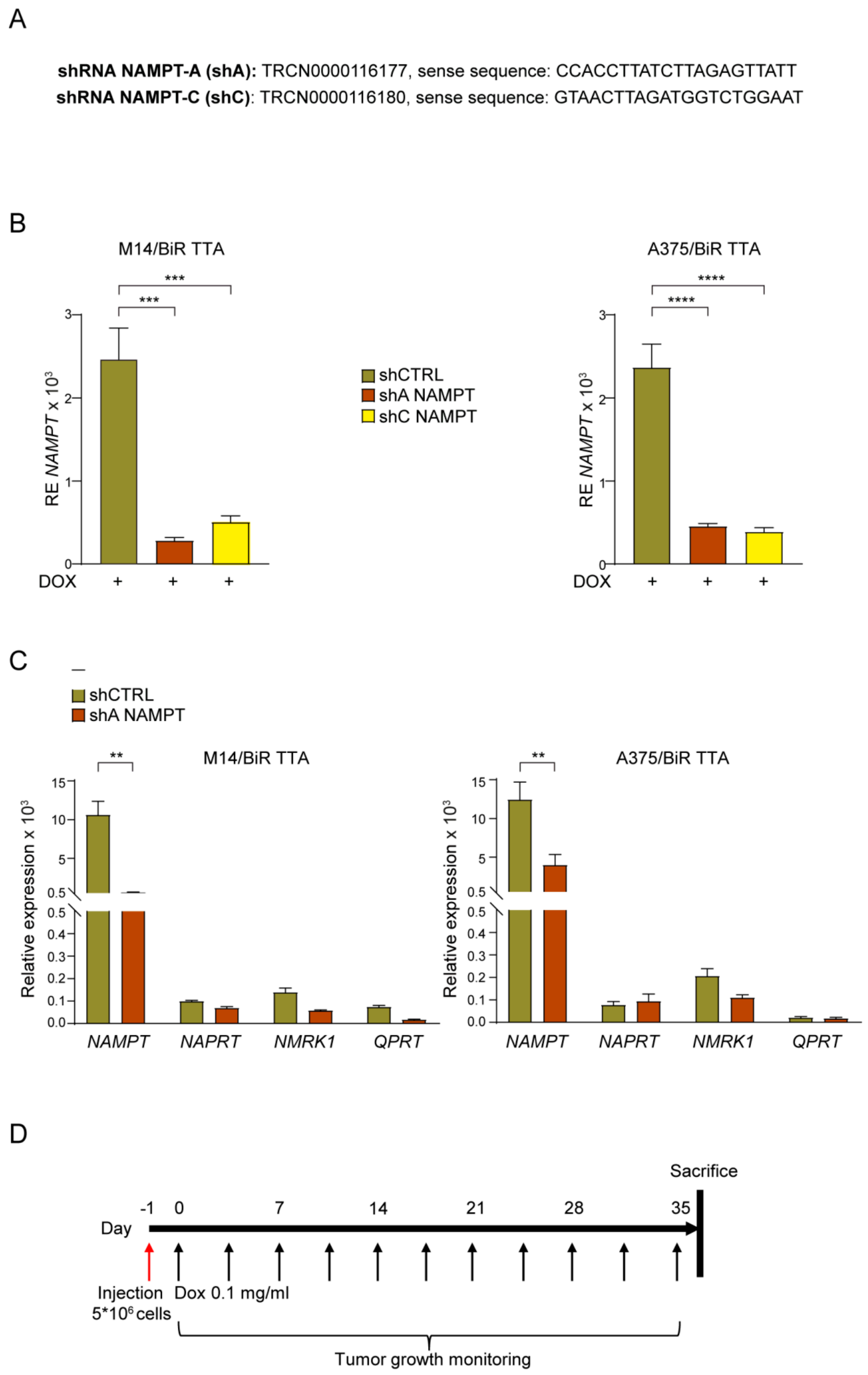

Figure S7. Inducible shRNA for NAMPT. (**A**) The two shRNA sequences for NAMPT (A–C) included in the lentiviral constructs, suitable for NAMPT gene silencing. (**B**) Histograms reporting the reduction of NAMPT mRNA expression levels, measured by qRT-PCR ($n = 4$, one-way Anova test) in M14/BiR TTA and A375/BiR TTA cell lines with a DOX-dependent inducible NAMPT silencing (using two different shRNAs NAMPT sequences A and C) in comparison with shCTRL cells. (**C**) Histograms showing cumulative RT-PCR analysis ($n = 3$, unpaired t test) of NBEs expression in M14/BiR TTA and A375/BiR TTA shCTRL and shA NAMPT after DOX treatment (1 µg/mL, 24 hours) to induce NAMPT silencing. Data in the Figure are presented as the mean ± SEM. Significance was represented as: * $p \leq 0.05$, ** $p \leq 0.01$, *** $p \leq 0.001$ and **** $p \leq 0.0001$.

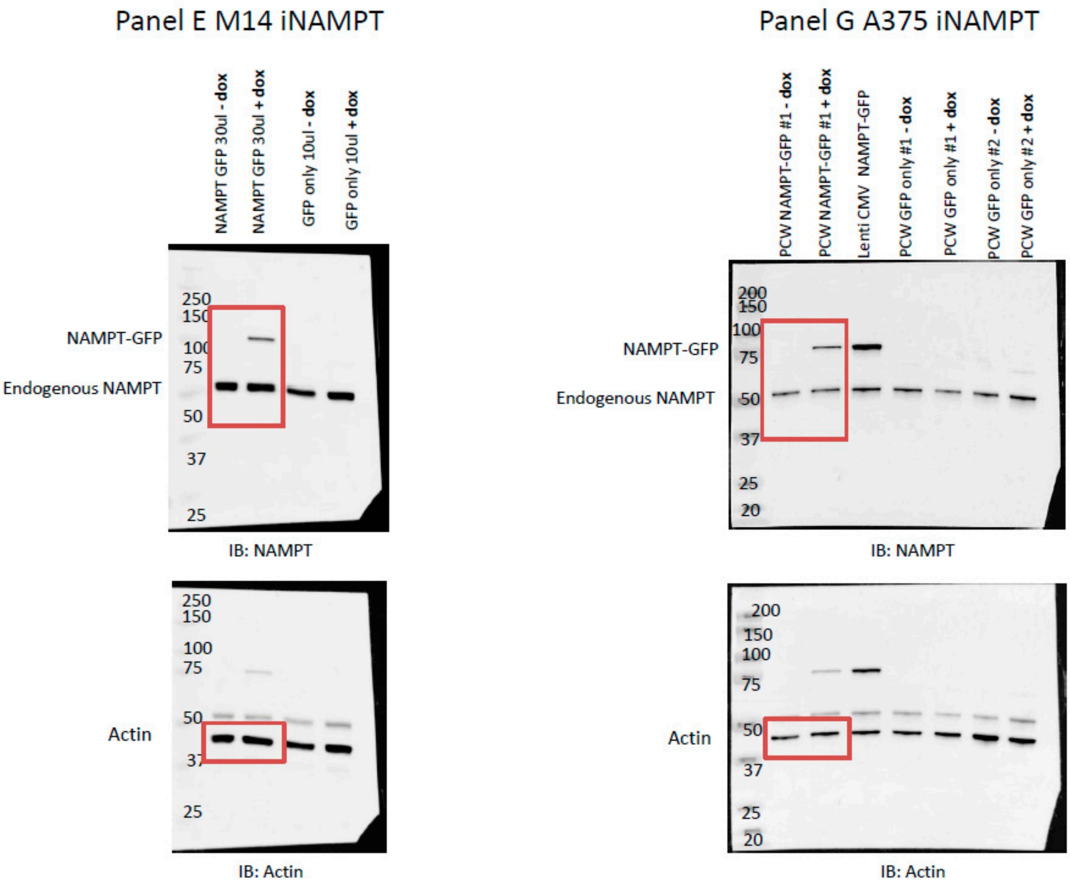

Figure S8. Uncropped Western Blot Images for Figure 2.

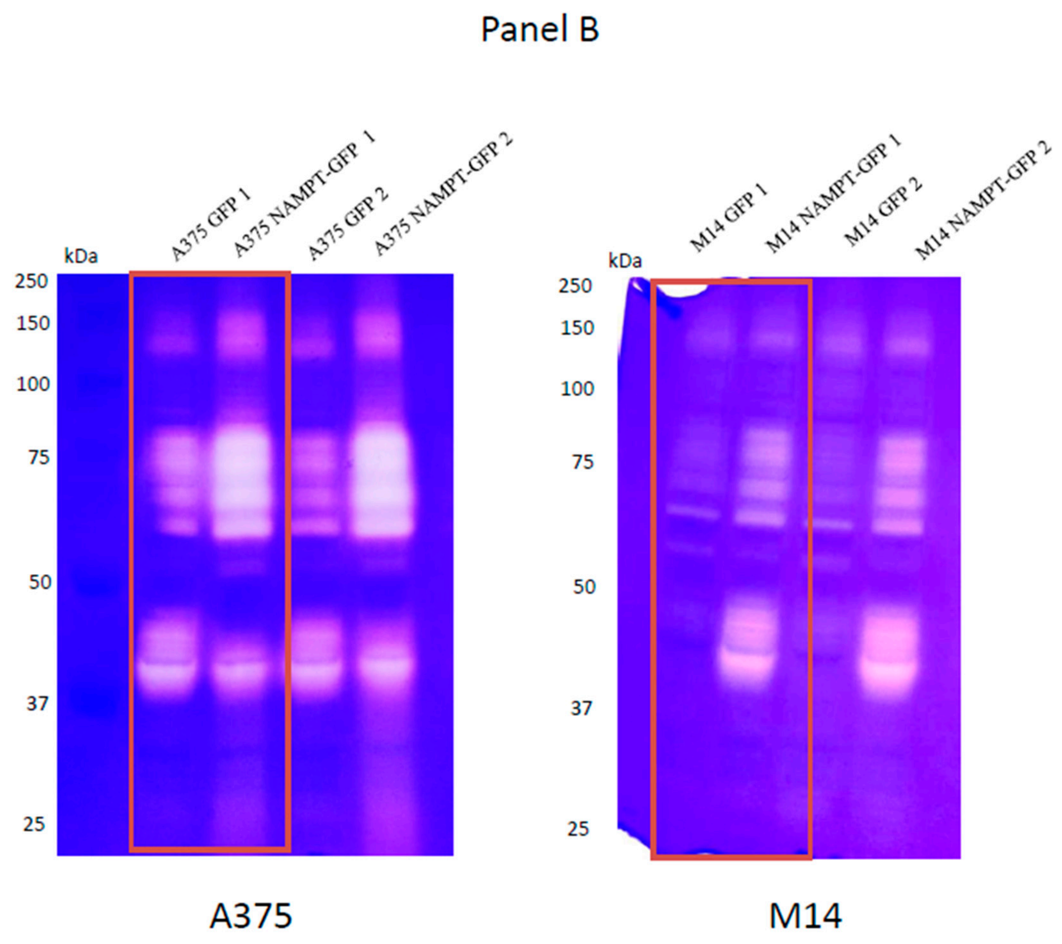

Figure S9. Uncropped Western Blot Images for Figure 3.

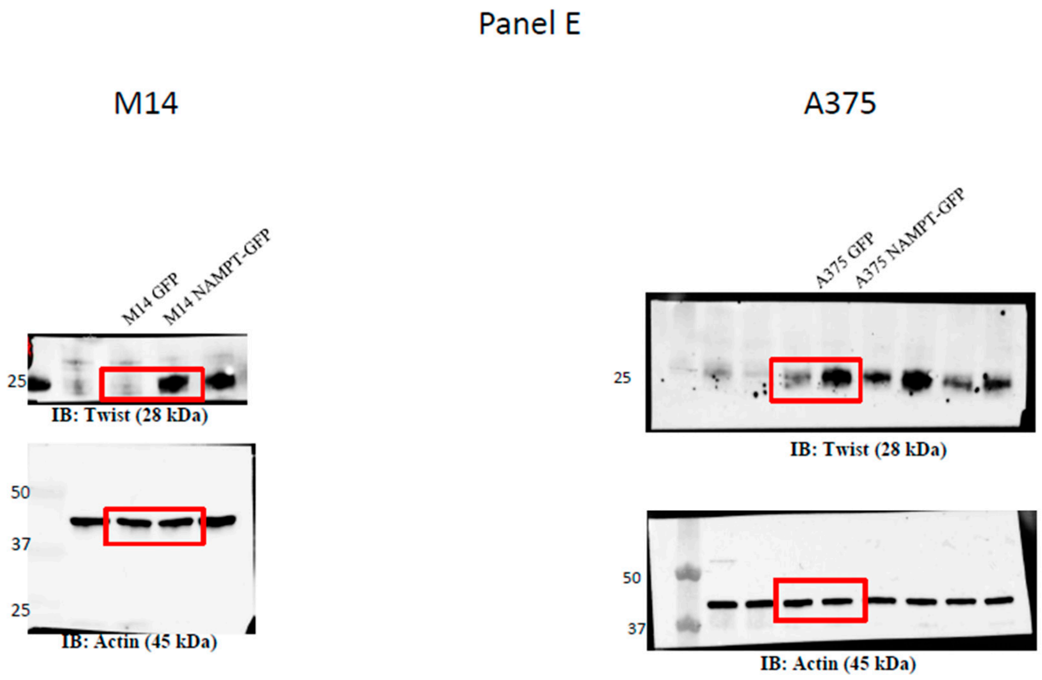

Figure S10. Uncropped Western Blot Images for Figure 3.

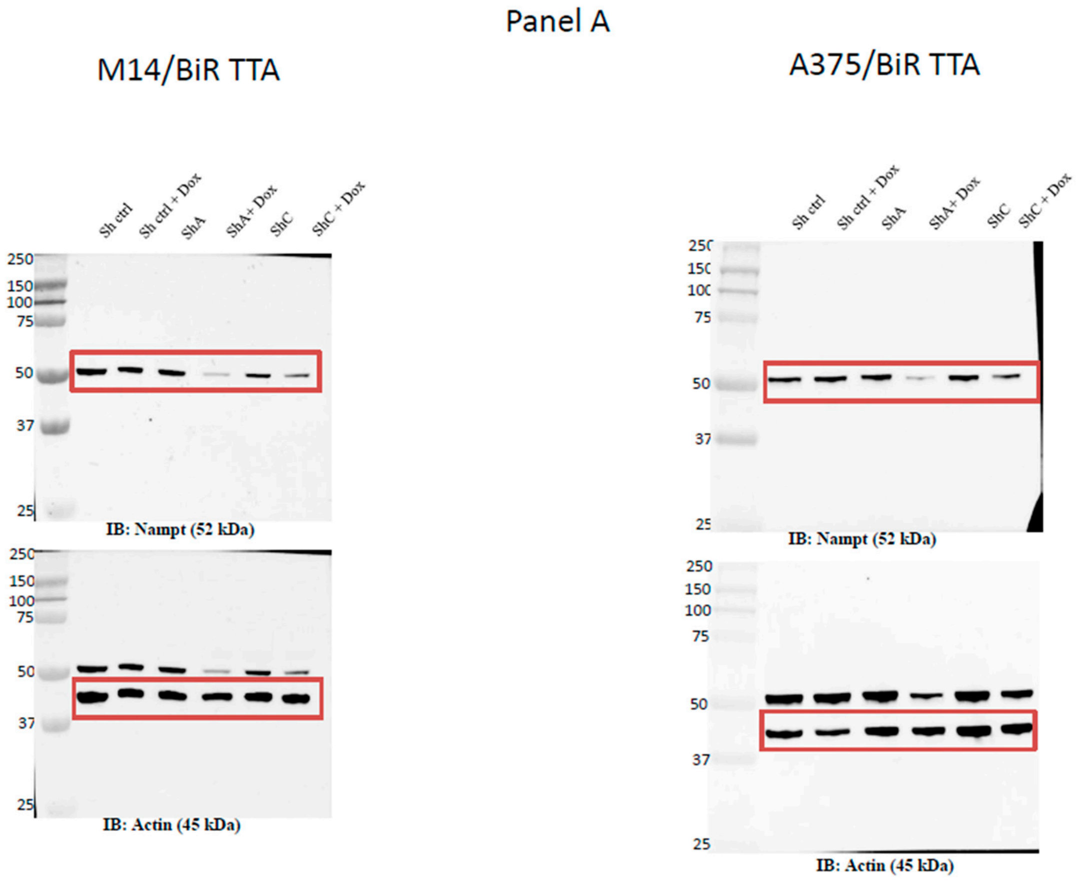

Figure S11. Uncropped Western Blot Images for Figure 6.

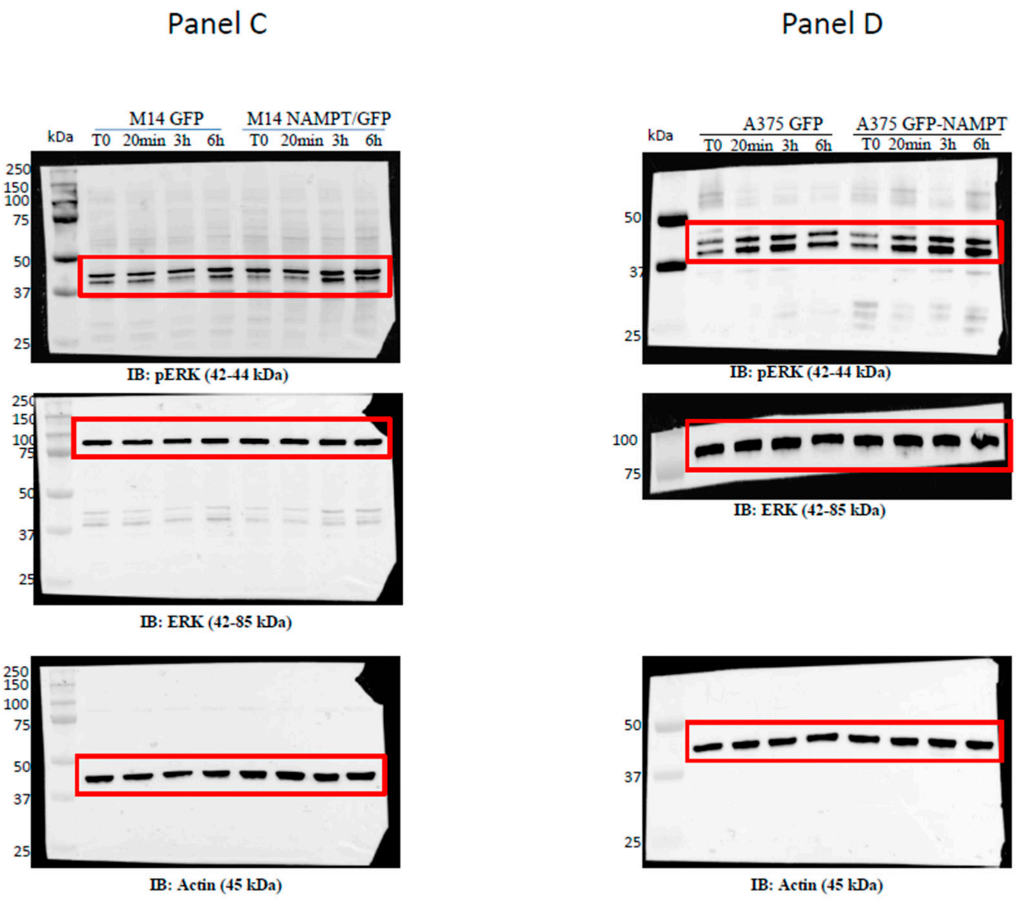

Figure S12. Uncropped Western Blot Images for Figure S2.

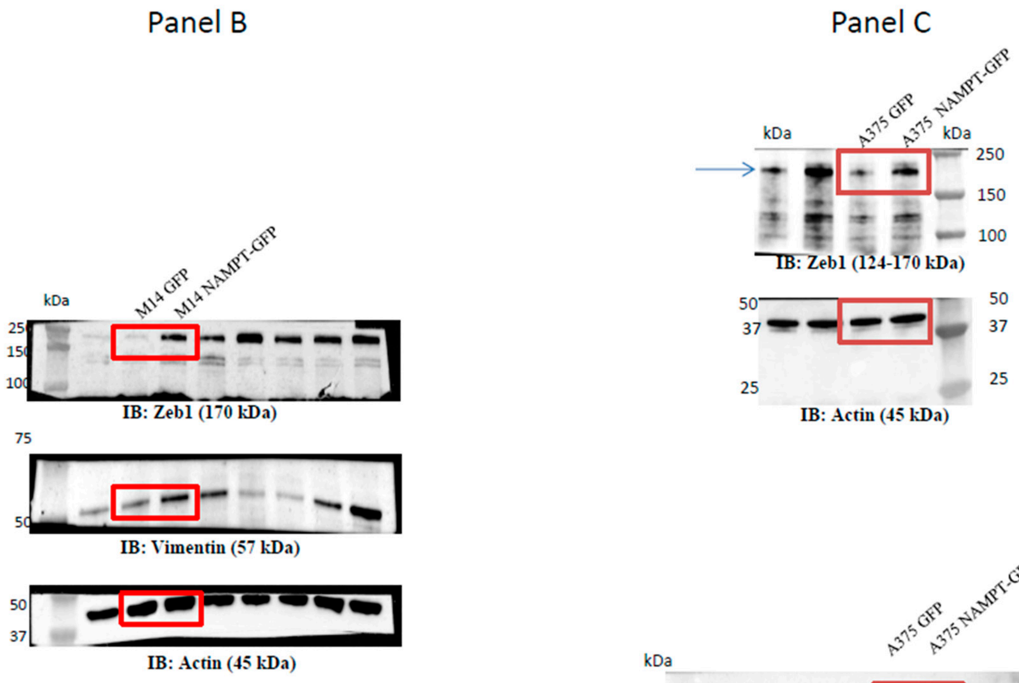

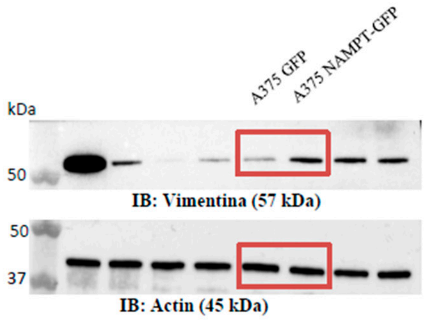

Figure S13. Uncropped Western Blot Images for Figure S4.

Panel E

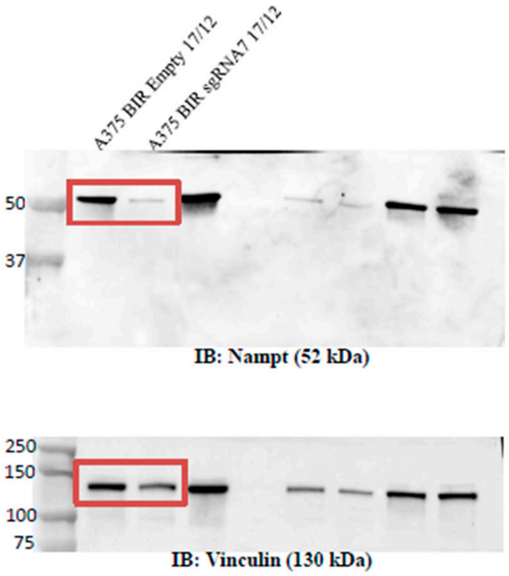

Figure S14. Uncropped Western Blot Images for Figure S6.

Publisher's Note: MDPI stays neutral with regard to jurisdictional claims in published maps and institutional affiliations.

© 2020 by the author. Licensee MDPI, Basel, Switzerland. This article is an open access article distributed under the terms and conditions of the Creative Commons Attribution (CC BY) license (http://creativecommons.org/licenses/by/4.0/).
